# TELEPHONE-BASED COGNITIVE ASSESSMENTS IN A LARGE, MULTISITE RCT: THE COSMOS-MIND STUDY

**DOI:** 10.1093/geroni/igz038.432

**Published:** 2019-11-08

**Authors:** Brad Caudle, Mark M Espeland, Stephen R Rapp, Sally A Shumaker, Debbie Pleasants, Laura D Baker

**Affiliations:** Wake Forest School of Medicine, Winston-Salem, North Carolina, United States

## Abstract

Identifying safe, affordable, and well-tolerated interventions that prevent or delay cognitive decline in older adults is of critical importance. There is growing evidence from basic science and small randomized trials that cocoa flavanols may provide protection against this decline. Funded by the NIA, COSMOS-Mind is an ancillary study of COSMOS and was designed to add cognitive outcomes to the parent study, a 2x2 factorial randomized controlled trial testing the effects of cocoa flavanols (600 mg/d) and a multivitamin with matching placebo on cardiovascular disease and cancer endpoints. A validated telephone-based protocol conducted at baseline and then annually for three years measures attention, memory, language, executive function, and global cognitive functioning in 2,262 women and men, ages 65 and older without insulin-dependent diabetes. Cases of mild cognitive impairment and Alzheimer’s and related dementias will be centrally adjudicated. For participants who score below a pre-specified threshold on a test of global cognition, a study partner is interviewed to obtain additional information regarding cognitive and functional status. With >5,000 interviews completed, this presentation will describe the cognitive battery, operational procedures used to ensure high data fidelity, and strategies employed that have maintained retention at >90%. Our experiences in COSMOS-Mind can inform the design and implementation of other large, multi-site RCTs and epidemiological studies. Telephone-based assessments of cognitive function are a cost-efficient method for assessing cognitive function.

